# Tocilizumab as a novel bridging therapy for surgery in dedifferentiated liposarcoma complicated by paraneoplastic leukemoid reaction, severe anemia, and thrombocytopenia: a case report

**DOI:** 10.3389/fonc.2026.1762067

**Published:** 2026-05-05

**Authors:** Jinjin Li, Ke Li, Shanshan Yu, Ya Li, Jiang Min, Zhen Huang, Zhengqiang Wei

**Affiliations:** 1 Department of Gastrointestinal Surgery, The First Affiliated Hospital of Chongqing Medical University, Chongqing, China; 2 Department of General Surgery, Chongqing Traditional Chinese Medicine Hospital, Chongqing, China; 3 Department of Pathology, The First Affiliated Hospital of Chongqing Medical University, Chongqing, China

**Keywords:** anemia, cytokine release syndrome, dedifferentiated liposarcoma, leukocytosis, paraneoplastic syndrome, thrombocytopenia, tocilizumab

## Abstract

**Background:**

Dedifferentiated liposarcoma (DDLPS) is a malignant mesenchymal tumor. While paraneoplastic leukemoid reaction (PLR) has been sporadically reported in DDLPS, its coexistence with severe anemia and thrombocytopenia, constituting a severe cytokine release syndrome (CRS), is exceedingly rare and presents a significant diagnostic and therapeutic challenge.

**Case presentation:**

A 56-year-old male with recurrent retroperitoneal well-differentiated liposarcoma presented with high fever, profound leukocytosis (WBC up to 43×10^9^/L), severe anemia (Hb 30 g/L), and thrombocytopenia (platelets 10×10^9^/L), which precluded immediate surgery. Extensive workup ruled out infection and hematological malignancies. The clinical picture was consistent with a tumor-induced CRS, evidenced by markedly elevated interleukin-6 (IL-6) and C-reactive protein (CRP). Following a multidisciplinary discussion, the patient received intravenous tocilizumab (an IL-6 receptor antagonist) and methylprednisolone. His fever resolved rapidly, and hematological parameters improved significantly, facilitating successful tumor resection. Postoperative pathology confirmed DDLPS. All paraneoplastic symptoms and laboratory abnormalities normalized after surgery. However, at the second follow-up, tumor recurrence was observed along with a marked elevation of the WBC, which again decreased following the subsequent surgical resection.

**Conclusion:**

This case highlights that DDLPS can trigger CRS, which manifests with clinical features mimicking severe infection and leading to critical cytopenias that complicate surgical management. IL-6 pathway blockade with agents such as tocilizumab may be an effective bridging therapy to stabilize the systemic inflammatory state and create a safe window for surgical intervention.

## Introduction

Dedifferentiated liposarcoma (DDLPS), a variant of liposarcoma, most frequently arises in the retroperitoneum and is characterized by high local aggressiveness and frequent recurrence (1, 2). Despite the expanding landscape of treatment modalities— including radiation, CDK4 inhibitor, targeted therapies, and immunotherapy— complete surgical resection remains the standard of care and the only potentially curative option (3–6). Paraneoplastic syndromes (PNS) refer to complex systemic clinical manifestations indirectly caused by a tumor, rather than by direct invasion or metastasis. PNS arises primarily through two mechanisms: first, the ectopic secretion of biologically active substances such as hormones, peptides, enzymes, or cytokines; and second, an immune-mediated response where anti-tumor antibodies cross-react with normal tissues. While common in lung, hematological, and renal malignancies, PNS are exceedingly rare in soft tissue sarcomas (7). Therefore, when a PNS manifests in association with DDLPS, it significantly complicates the diagnosis and management of the disease. Herein, we report a rare and instructive case of recurrent retroperitoneal DDLPS that presented with a severe PNS, characterized by a leukemoid reaction, profound anemia, and thrombocytopenia. We detail the successful preoperative management strategy utilizing tocilizumab- a targeted anti-cytokine therapy- as a bridge to safe surgical resection. This case provides valuable insights into the management of solid tumors presenting with similar systemic inflammatory manifestations.

## Case presentation

A 56-year-old male was admitted to our hospital with recurrent retroperitoneal liposarcoma 17 months after initial resection. His clinical history began with low back pain, which led to the discovery and subsequent resection of a 5 cm retroperitoneal mass at an external facility, pathologically confirmed well-differentiated liposarcoma. Two months later, local recurrence was detected via CT scan. The patient underwent CT-guided radiofrequency ablation, which was complicated by a left ureteral injury requiring stent placement. Two months before the current admission, the patient developed abdominal distension, and a repeat CT scan revealed a soft-tissue mass in the left mid-abdomen, suggestive of further recurrence (**Figure 1A**).

**Figure 1:**
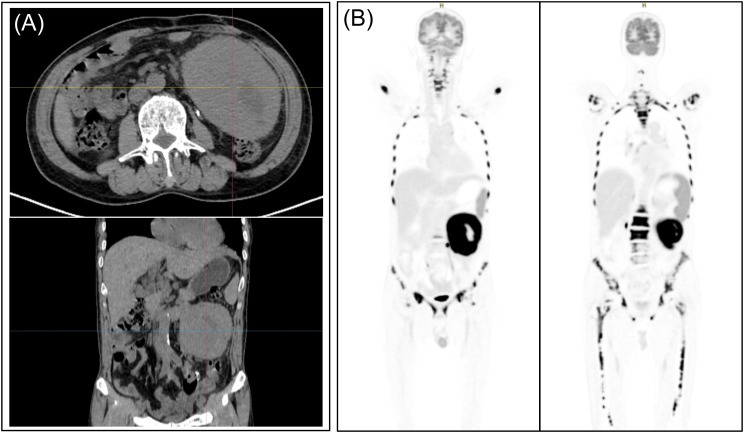
Imaging findings of the patient. **(A)** Abdominal CT upon admission reveals a soft tissue mass in the left abdomen, anterior to the descending colon. **(B)** Preoperative PET-CT demonstrates significantly increased radiotracer uptake in the left abdominal mass and diffusely increased radiotracer uptake throughout the systemic bone marrow.

Upon admission, the patient presented with recurrent high fever, peaking at 40 °C (Ref: 36.3-37.2°C). Laboratory investigations revealed a profound leukemoid reaction, with a white blood cell (WBC) count peaking at 43×10^9^/L (Ref: 3.5-9.5×10^9^/L). Concurrently, in the absence of gastrointestinal or other identifiable bleeding, his hemoglobin (Hb) dropped to 30 g/L (Ref: 130–175 g/L), and his platelet count decreased to 10×10^9^/L (100-300×10^9^/L). Despite treatments including blood and platelet infusions, recombinant human erythropoietin (rhEPO), and recombinant human thrombopoietin (rhTPO), these parameters failed to improve. Inflammatory markers were markedly elevated, including a C-reactive protein (CRP) of 304 mg/L (0-8mg/L) and interleukin-6 (IL-6) of 736.2 pg/mL (<5.3pg/mL) (**Figure 2**). This acute deterioration necessitated the postponement of surgery. To investigate a potential infectious source, we performed thoracic and abdominal CT along with multiple sets of cultures; however, no infectious etiology was identified. Despite the empirical administration of broad-spectrum antimicrobials— including ceftriaxone, ornidazole, piperacillin-tazobactam, cefoperazone-sulbactam, imipenem-cilastatin, vancomycin, linezolid, tigecycline, and voriconazole— the fever and hematological abnormalities persisted.

**Figure 2:**
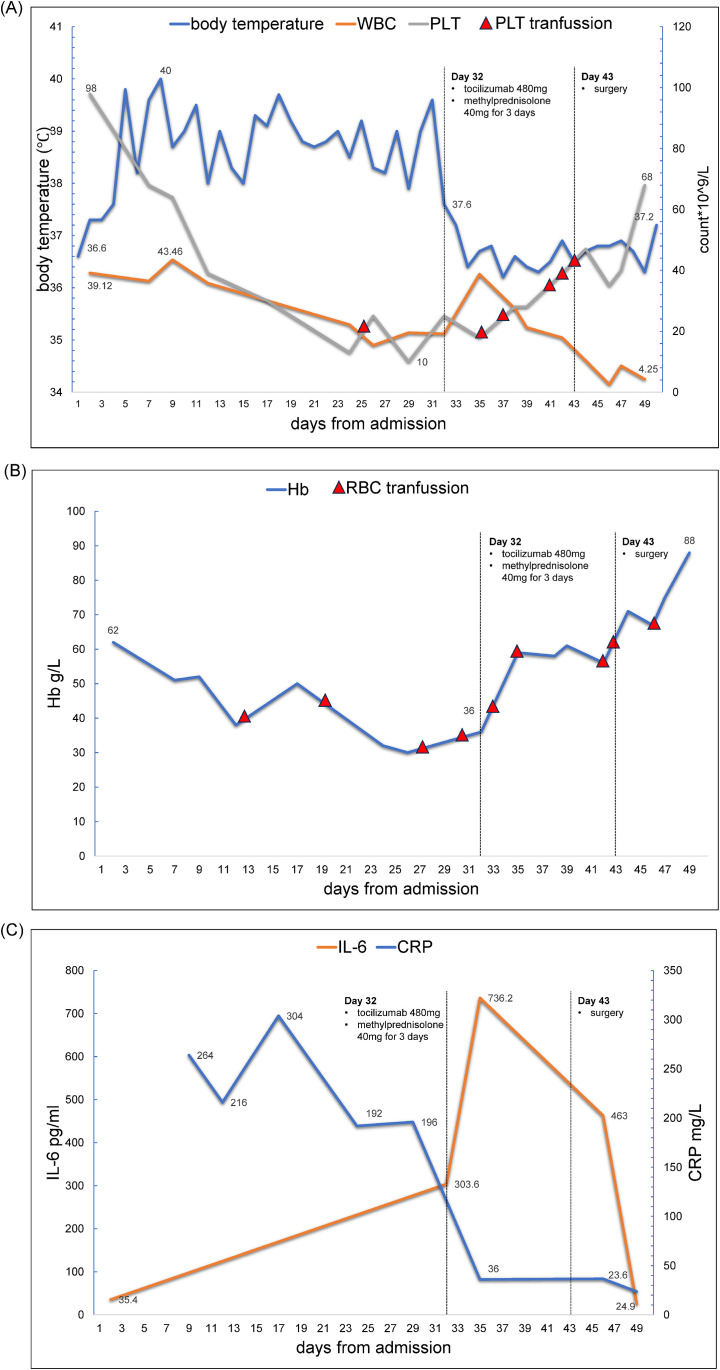
Line graphs showing the dynamics of the patient’s body temperature and hematological parameters. **(A)** After admission, the patient developed recurrent high fever and a markedly elevated WBC (3.5-9.5*10^9/L), while PLT (100-300*10^9/L) progressively decreased. Body temperature dropped immediately after initiation of drug therapy on Day 32, WBC began a downward trend, and PLT showed an upward trend; this trend became more pronounced after surgery on Day 43. **(B)** Hb (130-175g/L) levels gradually declined after admission, demonstrating an upward trend following Day 32 and Day 43. **(C)** IL-6 (0-0.5ng/ml) and CRP (0-8mg/L) levels exhibited a significant declining trend after surgery on Day 43. WBC, white blood cell; PLT, platelets; Hb, hemoglobin; RBC, red blood cell; IL-6, interleukin-6; CRP, C-reactive protein.

After excluding infection, we evaluated the patient for primary hematological malignancies. PET-CT demonstrated a large (12.6×8.0×11.8 cm), heterogeneously hypodense left mid-abdominal mass with intense radiotracer uptake (SUVmax 22.2), alongside diffusely increased skeletal uptake (SUVmax 13.3) (**Figure 1B**). Bone marrow biopsy revealed a hypercellular marrow with granulocytic hyperplasia but no evidence of aberrant blast proliferation. Extensive diagnostic testing— including hemolysis assays, autoantibody screening, and soluble interleukin-2 receptor (sCD25) levels— was unremarkable, effectively ruling out immune thrombocytopenia and hemophagocytic lymphohistiocytosis (HLH) (**Table 1**, **Supplementary Tables 1**, **2**).

**Table 1:** Other blood test results of the patient.

Items	Results	Reference range	Unit	Change
serum total cholesterol (TC)	2.33	2.8-5.2	mmol/L	↓
triglyceride (TG)	0.69	0.35-1.7	mmol/L	
sCD25	5683.93	0-600	pg/ml	↑
sCD40L	29.05	0-450	pg/ml	
sCD130	65897.28	19485-159951	pg/ml	
sTREM-1	184.69	0-115	pg/ml	↑
ferritin	611.7	30-400	ng/ml	↑
IL-1β	1.83	<12.40	pg/ml	
IL-2	3.15	<5.71	pg/ml	
IL-4	2.04	<3.00	pg/ml	
IL-5	1.77	<3.10	pg/ml	
IL-8	103.49	<53.09	pg/ml	↑
IL-10	10.56	<4.91	pg/ml	↑
IL-12 P70	2.5	<3.40	pg/ml	
IL-17	2.05	<20.60	pg/ml	
TNFα	2.05	<4.60	pg/ml	
IFN-α	1.82	<8.50	pg/ml	
IFN-γ	2.44	<7.42	pg/ml	
RET%	1.0	0.5-1.5	%	
RET#	13.90	24-84	10^9/L	↓
IRF	36.40	6.9-20.7	%	↓
Alb	28	35-50	g/L	↓
Tbil	6.2	3-22	umol/L	
CB	3.6	0-5	umol/L	
UCB	2.6	0-19	umol/L	
PT	13.7	11.0-14.5	s	
PTA	93.0	75-135	%	
APTT	38.7	28-44	s	
D-D	0.52	0-0.55	mg/L	
Fbg	8.05	1.8-3.8	g/L	↑
Coombs test	negative	negative	/	
fragmented red cells	0.2	0-1	%	
NK cells (CD3-CD16/56+) %	27.96	9.26-23.92	%	↑
serum amyloid protein A (SAA)	>400	<10.47	ug/ml	↑
Platelet-specific antibody detection	negative	N		

sCD25, soluble interleukin-2 receptor; sCD40L, soluble cluster of differentiation 40 ligand; sCD130, soluble glycoprotein 130; sTREM-1, soluble triggering receptor expressed on myeloid cells-1; IL, interleukin; TNFα, tumor necrosis factor-α; IFN, interferon; RET%, reticulocyte percentage; RET#, absolute reticulocyte count; IRF, immature reticulocyte fraction; Alb, Albumin; Tbil, total bilirubin; CB, conjugated bilirubin; UCB, unconjugated bilirubin; PT, prothrombin time; PTA, prothrombin time activity; APTT, activated partial thromboplastin time; D-D, d-dimer; Fbg, fibrinogen; NK cells, natural killer cells.

Following multidisciplinary (MDT) discussion, the patient was treated with intravenous tocilizumab (480 mg) and methylprednisolone (40 mg/day for 3 days). Fever resolved promptly after the first dose, the WBC count declined significantly, and there were no drug-related side effects. With continued transfusion support, the anemia and thrombocytopenia gradually improved, facilitating safe surgical intervention. We performed an en bloc resection of the tumor and the involved segment of the small intestine (**Figure 3A**). Despite an estimated blood loss of 1,400 mL, postoperative histopathological examination confirmed negative surgical margins. Postoperatively, WBC and CRP levels normalized without fever recurrence (**Figure 2**). The patient’s recovery was uneventful, and he was discharged on postoperative day 6. Pathology confirmed DDLPS with prominent inflammatory infiltration. Immunohistochemistry results were as follows: CD68 focal (+), S100 (+), MDM2 (+), CDK4 (–), CK (–), Ki-67 15% (+), CD34 vascular (+), DES partial (+), MelanA (–), HMB45 (–), and SMARCA4 (–). Genetic testing confirmed MDM2 gene amplification (MDM2/CEP12 ratio ≥2.0) (**Figure 3B**).

**Figure 3:**
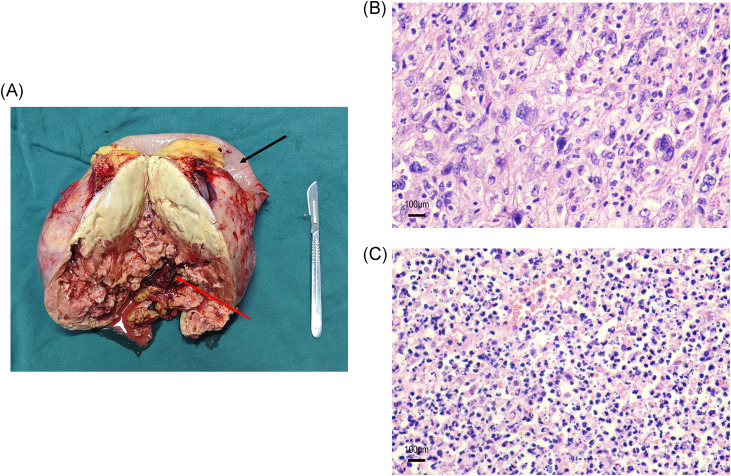
Macroscopic and microscopic pathological images of the tumor. **(A)** In the gross specimen image, the black arrow indicates tumor invades the small intestine, and the red arrow points to central necrosis within the tumor. **(B)** The tumor exhibits a disordered architecture with markedly atypical and pleomorphic cells. These cells feature enlarged, hyperchromatic nuclei with prominent nucleoli, and some display clear, vacuolated cytoplasm. Tumor giant cells are also present. **(C)** Focal areas show dense infiltration by both acute and chronic inflammatory cells.

At the two-month follow-up, blood counts remained normal with no radiologic evidence of recurrence. However, 223 days postoperatively, the patient returned with recurrent abdominal pain. CT imaging confirmed local recurrence at the primary site. Notably, the laboratory findings mirrored his initial presentation, including a marked leukemoid reaction, severe anemia, and elevated IL-6, accompanied by a low-grade fever (**Supplementary Figure 1**). Based on our prior experience, prompt radical resection was performed, including the tumor and involved segments of the sigmoid colon, small intestine, left kidney, and left ureter. Postoperatively, the WBC rapidly returned to normal limits (**Supplementary Figure 2**).

## Discussion

The presentation of liposarcoma accompanied by leukocytosis and fever has been documented in previous literature (8, 9). However, to our knowledge, this is the first reported case of DDLPS presenting with concomitant severe anemia and thrombocytopenia. While the majority of similar cases in the literature involve patients who could proceed directly to surgery, preoperative management in this instance was particularly challenging due to hematological contraindications that necessitated stabilization.

Tumor-associated leukemoid reaction has been observed in various solid malignancies, including lung, breast, and colorectal cancers, as well as sarcomas; it is formally recognized as paraneoplastic leukemoid reaction (PLR) (10). Currently, PLR remains a diagnosis of exclusion, established only after ruling out infection, the administration of granulocyte colony-stimulating factor (G-CSF), or the effects of corticosteroid therapy. PLR typically manifests as extreme leukocytosis (often >40×10^9^/L), predominantly neutrophilic (11). The underlying mechanism is primarily attributed to the secretion of G-CSF or G-CSF-like factors by tumor cells or tumor stromal cells, which stimulates myelopoiesis (12). In such patients, elevated G-CSF levels can often be detected in the serum or tumor tissue, and the condition generally resolves following effective antitumor therapy or surgical resection (8, 13). Leukocytosis caused by bone marrow metastasis has also been reported (10). In very rare cases of liposarcoma, bone marrow metastasis can occur, resulting in leukocytosis concomitant with bone marrow suppression (14). In the present case, PET-CT demonstrated diffuse and extensive radiotracer uptake throughout the bone marrow, a pattern inconsistent with the focal nature of typical bone marrow metastasis. Instead, this pattern likely reflects markedly heightened bone marrow metabolism. Previous studies indicate that G-CSF can induce widespread enhancement of bone marrow metabolic activity on PET scans, a phenomenon that subsides once the G-CSF stimulus is removed (15). Unfortunately, serum G-CSF testing was unavailable at our institution.

The emergence of severe anemia and profound thrombocytopenia was unexpected and presented a significant hurdle for surgical intervention. Given that surgical resection is the only potentially curative approach for retroperitoneal liposarcoma, and having excluded active bleeding and primary hematologic malignancies, we focused our investigation on the tumor’s systemic effects. The patient’s persistent high-grade fever, combined with markedly elevated serum IL-6 and CRP levels, indicated a profound systemic inflammatory state despite the lack of infectious evidence. Cytokines, acting as key mediators in inflammatory processes, can trigger cytokine release syndrome (CRS), also known as a cytokine storm (CS), when excessively released (16). While a standardized definition for CRS is lacking, it is characterized by immune cell overactivation leading to a massive release of interleukins, interferons, and chemokines (17, 18). If infection is ruled out, a diagnosis of CRS can be established based on recurrent and significantly elevated cytokine levels (19). While infection and sepsis are the most common triggers, CRS is also frequently observed in the context of CAR-T cell therapy or therapeutic monoclonal antibodies, such as anti-CD3, anti-CD52, and anti-CD20 antibodies, as well as the CD28 superagonist TGN1412 (20, 21). Crucially, solid tumors themselves can induce CRS; specific tumor cells or their microenvironment stroma can secrete vast quantities of cytokines, driving a hyper-inflammatory state. This cytokine network plays a pivotal role in cancer-associated inflammation, regulating both host and malignant cell behaviors within the tumor microenvironment (22, 23).

The patient developed severe preoperative anemia that worsened progressively despite intermittent treatment with rhEPO and blood transfusions. Beyond the tumor’s consumptive effects, systemic inflammation is a major driver of anemia in such cases (24). The core pathophysiology involves inflammation-induced upregulation of cytokines, particularly IL-6, which increases levels of hepcidin, the key regulator of iron homeostasis. Elevated hepcidin inhibits intestinal iron absorption and promotes iron sequestration within the reticuloendothelial system, leading to iron-restricted erythropoiesis. Furthermore, the inflammatory state can shorten red blood cell survival, blunt the compensatory erythropoietin response to anemia, and directly suppress erythroid differentiation via inflammatory mediators, collectively contributing to the manifestation of “anemia of inflammation” with its disease-specific characteristics. Diagnosis relies on normocytic normochromic anemia, evidence of systemic inflammation (elevated ESR/CRP), and iron restriction parameters (transferrin saturation <20% with ferritin >100 μg/L) without systemic iron deficiency (25).

Retroperitoneal tumor resection carries a high risk of major hemorrhage, which is substantially exacerbated by concurrent severe thrombocytopenia. While the etiology of thrombocytopenia in this context is multifactorial, CRS is a significant contributor (26). Similar to the mechanisms observed in sepsis and COVID-19-associated CRS, the pathophysiology likely involves impaired platelet production, cytokine-mediated destruction, splenic sequestration, and immune-mediated clearance, all of which elevate surgical risk (27).

Based on the above analysis, the constellation of clinical manifestations in this patient was ultimately attributed to a tumor-induced CRS— a form of paraneoplastic syndrome termed “paraneoplastic inflammatory syndrome (PIS)” by Blay et al. (28). Although CRS involves a complex network of cytokines, IL-6 has been identified as a central regulatory node (20, 21). Consequently, anti-inflammatory therapy targeting the IL-6 pathway is a logical strategy. Tocilizumab, a humanized monoclonal antibody against the IL-6 receptor, inhibits the inflammatory cascade by blocking both membrane-bound and soluble receptors. While currently approved for rheumatoid arthritis and CAR-T cell-induced CRS (29–32). During the COVID-19 pandemic, IL-6 inhibition therapy was used to manage CRS triggered by viral infection, demonstrating favorable efficacy (33, 34). A study investigating tocilizumab for PIS in solid tumors included 35 patients, 10 of whom had DDLPS. Treatment resulted in fever resolution in 97% of cases and a significant increase in average hemoglobin levels; notably, some patients who were initially ineligible for radical surgery became surgical candidates following treatment (28). Although tocilizumab effectively alleviates systemic symptoms, its impact on the tumor’s biological behavior remains unclear. In responsive patients, fever typically resolves within hours. If the response is suboptimal, secondary immunosuppressants such as corticosteroids— which are second-line for CAR-T associated CRS— can be added and often tapered rapidly (21). Notably, due to various constraints, we were unable to assess the expression of cytokines such as G-CSF and IL-6 within the tumor specimens. Consequently, it remains undetermined whether the markedly elevated serum IL-6 levels originated directly from the tumor cells or were secreted by host immune cells. Future studies are encouraged to address this limitation to further elucidate the underlying mechanism.

Notably, all paraneoplastic symptoms and hematological abnormalities normalized following tumor resection. It has been reported that these parameters can resurge upon tumor recurrence, sometimes preceding radiographic evidence (9). This phenomenon was corroborated by the laboratory findings before our patient’s second surgery, suggesting that these inflammatory markers may serve as sensitive references for monitoring tumor recurrence, holding significant clinical implications, especially for sarcomas, which often lack specific tumor markers.

## Conclusion

The PNS observed in this patient is considered to result from aberrant tumor secretion of G-CSF and the induction of a CRS. In oncological patients presenting with recurrent fever, leukemoid reactions, severe anemia, or thrombocytopenia, clinicians should maintain a high index of suspicion for tumor-induced PNS once common etiologies, such as infection, have been excluded. If the patient’s systemic condition temporarily precludes surgery, inflammatory cytokine blockers (e.g., tocilizumab) or glucocorticoids may be considered to control the systemic inflammation and improve the clinical condition, thereby creating a safe window for curative surgery.

## Data Availability

The original contributions presented in the study are included in the article/**Supplementary Material**. Further inquiries can be directed to the corresponding authors.
