# Supplementary figures and images for: Tocilizumab as a novel bridging therapy for surgery in dedifferentiated liposarcoma complicated by paraneoplastic leukemoid reaction, severe anemia, and thrombocytopenia: a case report
Source: Front Oncol. 2026 May 5;16:1762067. doi: 10.3389/fonc.2026.1762067 (PMC13183568; doi:10.3389/fonc.2026.1762067)

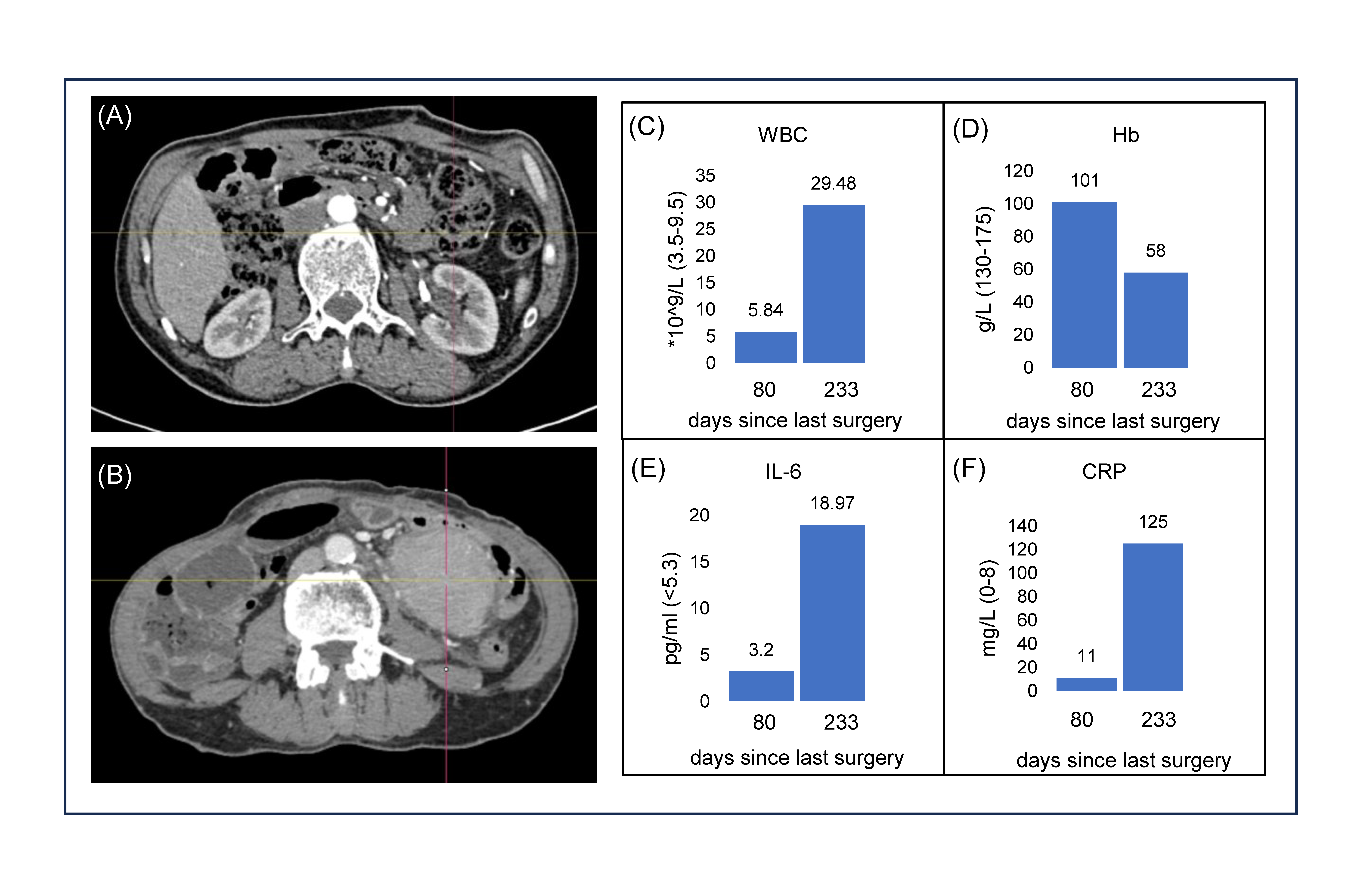

Supplement: Supplementary file 1 [file Image1.tif]

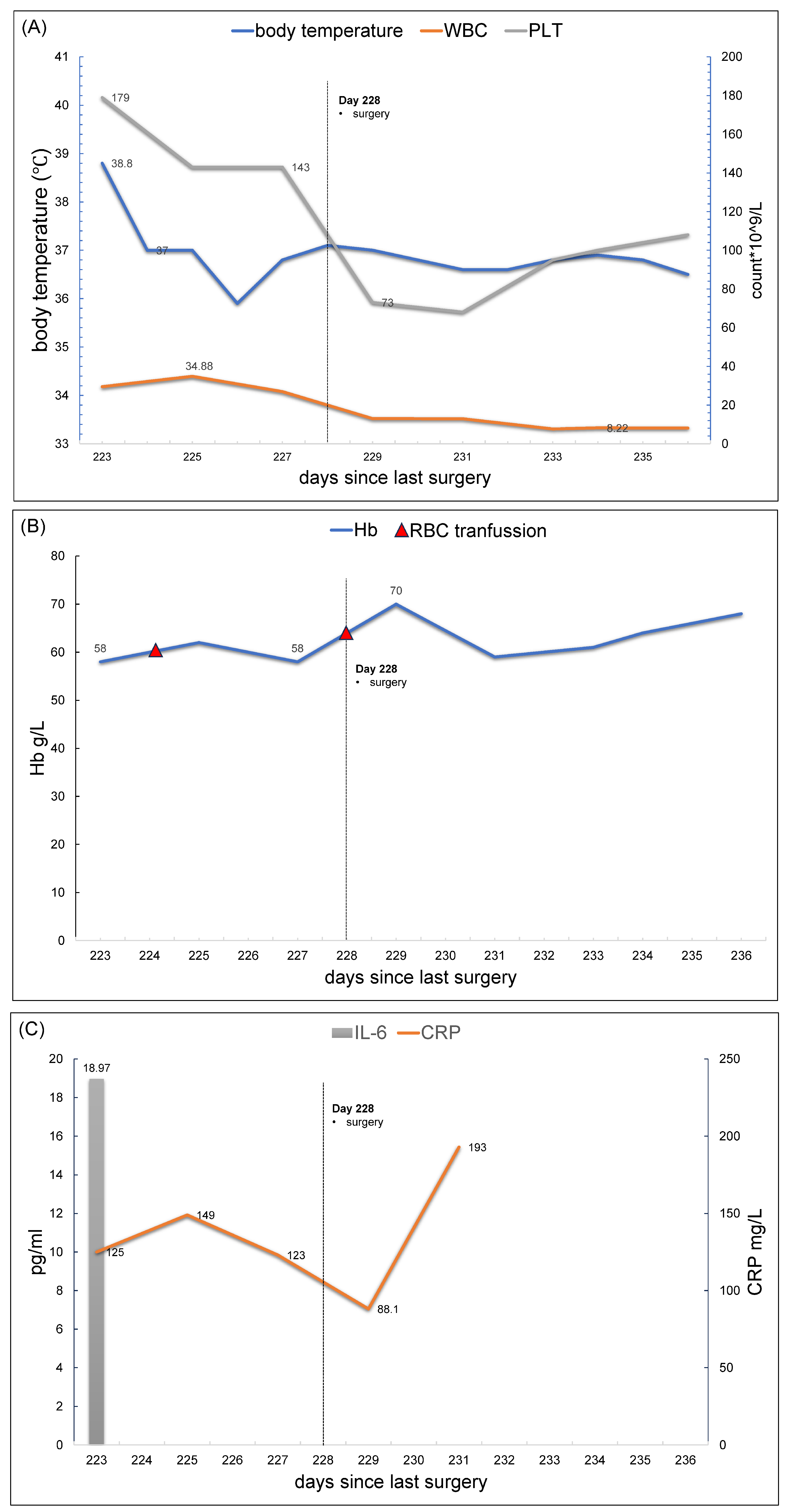

Supplement: Supplementary file 2 [file Image2.tif]
