# Supplementary material for: Tocilizumab as a novel bridging therapy for surgery in dedifferentiated liposarcoma complicated by paraneoplastic leukemoid reaction, severe anemia, and thrombocytopenia: a case report
Source: Front Oncol. 2026 May 5;16:1762067. doi: 10.3389/fonc.2026.1762067 (PMC13183568; doi:10.3389/fonc.2026.1762067)
Supplement: Supplementary file 3 [file Table1.docx]

Supplementary Table 1 Antinuclear Antibody Profile (ANA Profile)

| items | results | reference range | unit | change |
| --- | --- | --- | --- | --- |
| ANA (IIF) | Negative | Negative (< 1:100) | - |  |
| u1-nRNP | < 2.0 | < 20.0 | RU/ml |  |
| Sm | < 2.0 | < 20.0 | RU/ml |  |
| SSA | < 2.0 | < 20.0 | RU/ml |  |
| Ro-52 | < 2.0 | < 20.0 | RU/ml |  |
| SSB | < 2.0 | < 20.0 | RU/ml |  |
| Scl-70s | < 2.0 | < 20.0 | RU/ml |  |
| PM-Scl | < 2.0 | < 20.0 | RU/ml |  |
| Jo-1 | < 2.0 | < 20.0 | RU/ml |  |
| CENP-B | < 2.0 | < 20.0 | RU/ml |  |
| PCNA | < 2.0 | < 20.0 | RU/ml |  |
| ds-DNA | < 1.0 | < 10.0 | IU/ml |  |
| AnuA | < 2.0 | < 20.0 | RU/ml |  |
| Histone | < 2.0 | < 20.0 | RU/ml |  |
| rRNP | < 2.0 | < 20.0 | RU/ml |  |
| AMA-M2 | < 2.0 | < 20.0 | RU/ml |  |
|  |  |  |  |  |

ANA (IIF), antinuclear antibody (indirect immunofluorescence); u1-nRNP, anti-u1 ribonucleoprotein antibody; Sm, anti-Smith antibody; SSA, anti-Sjögren's syndrome-related antigen A antibody; Ro-52, anti-Ro-52 antibody; SSB, anti-Sjögren's syndrome-related antigen B antibody; Scl-70s, anti-Scl-70 (Topoisomerase I) antibody; PM-Scl, anti-Polymyositis-Scleroderma antibody; Jo-1, anti-Jo-1 (Histidyl-tRNA synthetase) antibody; CENP-B, anti-Centromere Protein B antibody; PCNA, anti-Proliferating Cell Nuclear Antigen antibody; ds-DNA, anti-double-stranded DNA antibody; AnuA, anti-Nucleosome antibody; Histone, anti-Histone antibody; rRNP, anti-ribosomal P protein antibody; AMA-M2, anti-Mitochondrial Antibody M2 subtype.

Supplementary Table 2 Antineutrophil Cytoplasmic Antibody Profile (ANCA Profile)

| items | results | reference range | unit | change |
| --- | --- | --- | --- | --- |
| cANCA (IIF) | Negative | Negative (< 1:10) | - |  |
| pANCA (IIF) | Negative | Negative (< 1:10) | - |  |
| aANCA (IIF) | Negative | Negative (< 1:10) | - |  |
| MPO_IgG | < 1.50 | 0.00 - 20.00 | AU/ml |  |
| PR3_IgG | < 2.00 | 0.00 - 20.00 | AU/ml |  |
| GBM_IgG | < 2.00 | 0.00 - 20.00 | AU/ml |  |
|  |  |  |  |  |

cANCA (IIF), cytoplasmic antineutrophil cytoplasmic antibody (indirect immunofluorescence); pANCA (IIF), perinuclear antineutrophil cytoplasmic antibody (indirect immunofluorescence); aANCA (IIF), atypical antineutrophil cytoplasmic antibody (indirect immunofluorescence); MPO_IgG, anti-myeloperoxidase IgG antibody; PR3_IgG, anti-proteinase 3 IgG antibody; GBM_IgG, anti-glomerular basement membrane IgG antibody.

Supplementary Figure 1 Follow-up imaging and laboratory results. (A) Computed tomography (CT) at the first follow-up (postoperative day 80) showing no evidence of local recurrence. (B) CT at the second follow-up (postoperative day 233) revealing local recurrence at the primary site. (C–F) Comparative levels of WBC (Ref: 3.5-9.5*10^9/L) count, Hb (Ref: 130-175g/L), IL-6 (Ref: 0-0.5ng/ml), and CRP(Ref: 0-8mg/L) at the first and second follow-ups.

WBC, white blood cell; Hb, hemoglobin; IL-6, interleukin-6; CRP, C-reactive protein.

Supplementary Figure 2 Postoperative dynamics of laboratory parameters following the second surgery. (A) Marked decline in WBC (Ref: 3.5-9.5*10^9/L) count postoperatively, consistent with the initial surgical outcome; (B) Trends in Hb (Ref: 130-175g/L) levels; (C) Longitudinal changes in IL-6 (Ref: 0-0.5ng/ml) and CRP (Ref: 0-8mg/L) levels.

WBC, white blood cell; Hb, hemoglobin; IL-6, interleukin-6; CRP, C-reactive protein.
